# The effects of compound probiotics on production performance, rumen fermentation and microbiota of *Hu* sheep

**DOI:** 10.3389/fvets.2024.1440432

**Published:** 2024-10-31

**Authors:** Lijun Wang, Zhanqi Lv, Xiaodong Ning, Zhiguang Yue, Ping Wang, Chaoqi Liu, Sanjun Jin, Xinxin Li, Qingqiang Yin, Qun Zhu, Juan Chang

**Affiliations:** ^1^College of Animal Science and Technology, Henan Agricultural University, Zhengzhou, China; ^2^Henan Technical Institute, Zhengzhou, China; ^3^Henan Anjin Biotechnology Co., Ltd., Xinxiang, China; ^4^Henan Delin Biological Product Co., Ltd., Xinxiang, China

**Keywords:** *Hu* sheep, compound probiotics, growth performance, immune function, rumen bacteria

## Abstract

Fungal probiotics have the potential as feed additives, but less has been explored in ruminant feed up to date. This study aimed to determine the effect of compound probiotics (CPs) with *Aspergillus oryzae* 1, *Aspergillus oryzae* 2 and *Candida utilis* on *Hu* sheep’s growth performance, rumen fermentation and microbiota. A total of 120 male *Hu* sheep, aged 2 months and with the body weight of 16.95 ± 0.65 kg were divided into 4 groups. Each group consisted of 5 replicates, with 6 sheep per replicate. Group A was the control group fed with the basal diet. Group B, C and D was supplemented with the basal diet by adding 400, 800 and 1,200 grams per ton (g/t) CPs, respectively. The feeding trial lasted for 60 days after a 10-day adaptation period. The results showed that the average daily gain (ADG) of sheep in the CPs groups were significantly higher, the feed/gain were significantly lower than those in group A in the later stage and the overall period. The addition of CPs increased the economic benefit. The levels of CD4^+^ and the CD4^+^/CD8^+^ ratio in the CPs groups were higher than those in Group A. The levels of GSH, IgG, IL-2, IL-6, and IFN-*γ* in group C were significantly elevated compared with group A. Group B showed a significant increase in rumen NH_3_-N and cellulase activity. There was no difference in VFAs content between group A and group B, however, with the increasing addition of CPs, the butyric acid and isobutyric acid content tended to decrease. The rumen microbiota analysis indicated that the CPs addition increased the *Firmicutes* and *Proteobacteria* abundances, decreased the *Bacteroidetes* abundance. The correlation analysis showed that *Prevotella* was negatively correlated with ADG, and the addition of 400 CPs in group B reduced *Prevotella*’s relative abundance, indicating CPs increased sheep growth by decreasing *Prevotella* abundance. The CPs addition reduced caspase-3, NF-κB and TNF-*α* expression in liver, jejunum and rumen tissues. In conclusion, the addition of CPs increased the sheep production performance, reduced inflammation, improved rumen and intestinal health. Considering the above points and economic benefits, the optimal addition of CPs as an additive for *Hu* sheep is 800 g/t.

## 1 Introduction

Antibiotics, beyond controlling pathogenic microorganisms, also affect many beneficial microorganisms, causing disturbances to the balance of the gastrointestinal microbiota (1). The abused of antibiotics can result in the development of drug-resistant bacteria and the presence of antibiotic residues in livestock products, which may subsequently enter the human body through the food chain and adversely affect human health (2). Adding antibiotics to feed is already banned, therefore, it is more important to study the substitutes of antibiotics. Probiotics are a class of active microorganisms that can be beneficial to the host by colonize the host’s body and alter the balance of gut microbiota (1). In recent years, probiotics have gained increasing attention as alternatives to antibiotics due to their ability to enhance animal productivity (3), inhibit pathogenic microorganism colonization (4), and maintain the balance of microflora of the digestive tract of the host (5). Probiotic preparation on the market includes bacteria, fungus and yeast (6). In the past, the application in animal husbandry mainly focused on bacteria and yeast, while little attention has been paid on to fungal probiotics supplementation in ruminants.

The fungal probiotic field is one of the developing fields nowadays (7). Numerous studies have described the role of *Aspergillus oryzae* as one of the most common probiotics and has been widely used as a feed additive in ruminant production (8–10). *Aspergillus oryzae* enhances feed dry matter degradation, reduces inflammation, and increases the energy supply of total volatile fatty acids (VFAs) in the rumen (8). Additionally, *Aspergillus oryzae* plays a role in the rumen and hindgut (11). *Candida utilis* cell wall contains *β*-glucan, glucomannan and mannoprotein (12), which have been shown to improve intestinal health, inhibit cellular DNA damage, antioxidant, antimutagenic and antitoxic activities (13). Many studies have shown that *Candida utilis* used in feed can improve animal production performance, antioxidant capacity, immune function and increase the villus height of the jejunum and ileum (14–16).

Even though the effectiveness of individual *Aspergillus oryzae* and *Candida utilis* has been studied, most studies are limited to a single strain. Still, single strains have problems such as poor stability and insignificant application effect (12). This study aimed to investigate the effect of combined additive with *Aspergillus oryzae* and *Candida utilis* on growth performance, antioxidant capacity, immunity, ruminal fermentation and microbial diversity of *Hu* sheep. It will provide scientific basis for the application of compound probiotics in ruminant production.

## 2 Materials and methods

### 2.1 Animal ethics statement

All animal experimental procedures were approved by the Animal Care and Use Committee of Henan Agricultural University (Approval number: HENAU-2021-025).

### 2.2 Materials preparation

*Aspergillus oryzae* 1 (CGMCC5817) used in this study was isolated from a cow rumen, and identified by 26S rDNA, it was preserved in China General Microbiological Culture Collection Center (CGMCC)*. Aspergillus oryzae* 2 (CGMCC3.0496) and *Candida utilis* (CGMCC2.0615) were obtained from CGMCC. They were incubated according to the published protocols to make the probiotics powder (17). Based on the previous results obtained with response surface regression design *in vitro* in our laboratory. The compound probiotics (CPs) were prepared by the optimal ratio of *Aspergillus oryzae* 1, *Aspergillus oryzae* 2 and *Candida utilis* with 1 × 10^4.45^, 1 × 10^5^ and 1 × 10^5^ colony-forming units (CFU)/ g compound probiotics powder, respectively.

### 2.3 Animals, diets, and experimental design

This experimental design was approved by the Sanmu Lvyuan Livestock Breeding Co., Ltd. (Dengfeng, China). A total of 120 male *Hu* sheep, aged 2 months and with the body weight of 16.95 ± 0.65 kg were divided into 4 groups. Each group consisted of 5 replicates, with 6 sheep per replicate. Group A was fed basal diet (control group); Group B was fed basal diet with 400 g/t CPs; Group C was fed basal diet with 800 g/t CPs; and Group D was fed basal diet with 1,200 gram per ton (g/t) CPs. The feeding experiment duration was 70 days, which included a 10-day adaptation period. The pre-trial period lasted 30 days (1–30 day), and the post-trial period for 30 days (31–60 day). The sheep were fed twice daily at 08:00 and 16:00. The amount of feed offered to each pen, as well as leftover feed, was recorded daily. They were provided access to clean, fresh water *ad libitum*. The diet was formulated according to Nutrition Requirement of Meat Sheep in China (NY/T 816–2021) to meet the nutritional requirements. Ingredients and chemical composition of the total mixed diet are shown in Table 1.

**Table 1:** Feed composition and nutrient levels of basal diets (%, dry matter basis).

Feed composition	Content (%)	Nutrient levels^a^	Content
Corn meal	20.40	Metabolic energy (MJ/kg)	10.14
Soybean meal	12.80	Crude protein (%)	14.09
Wheat bran	3.56	Ether extract (%)	5.06
Corn silage	10.00	Neutral detergent fiber (%)	50.30
Peanut Straw	50.00	Acid detergent fiber (%)	22.24
NaCl	0.28	Calcium (%)	1.09
NaHCO3	0.64	Phosphorus (%)	0.43
Limestone	0.24	Ash (%)	9.06
CaHPO4	0.08		
Premix compound*	2.00		
Total	100.00		

* Composition of premix compound (per kilogram of the diet): Cu (copper sulfate) 16.0 mg, Fe (ferrous sulfate) 60.0 mg, Mn (manganese sulfate) 59.0 mg, Zn (zinc sulfate) 120.0 mg, I (calcium iodate) 1.4 mg, Se (sodium selenite) 0.10 mg, Co (cobalt chloride) 0.03 mg, Vitamin A (VA) 14,400 IU, VD_3_ 4,400 IU, VE 30 mg.

a Metabolic energy was a calculated value, while others was measured.

### 2.4 The determination of growth performance

Body weight for each replicate were recorded on day 1, 30, and 60, respectively, to calculate the average daily gain (ADG), average daily feed intake (ADFI) and feed/gain (F/G) for days 1 to 30, 31 to 60, and 1 to 60 (1to 60, the entire test period). ADG, ADFI, and F/G were calculated as follows:


$$\begin{array}{l} ADG=\left(\mathrm{finish weight}–\mathrm{start weight}\right)/\mathrm{age}\;\left(\mathrm{days}\right),\\ \mathrm{ADFI}=\left(\begin{array}{l} \mathrm{provide feed amount}-\\ \mathrm{residual feed amount} \end{array}\right)/\mathrm{number of sheep},\\ F/G=\mathrm{average daily feed intake}/\\ \mathrm{average daily gain} \end{array}$$

### 2.5 The determination of digestibility

On days 28 and 58, fecal samples were collected from five sheep in each group for three consecutive days. Total feces from each sheep were collected, weighed, mixed, and sampled twice daily (0900 and 1700 h). Representative samples (100 g each) were mixed with 10 mL of 10% (wt/wt) hydrochloric acid and stored in a sterile plastic bag at −20°C for further analysis. Some fecal samples were dried at 65°C for 24 h and mashed to determine apparent nutrient digestibility. The apparent nutrient digestibility was calculated according to formula:


$$\begin{array}{l} \mathrm{Nutrient}\;\\ \mathrm{digestibility}\;\left(\%\right)=\left[\left(\begin{array}{l} \mathrm{Nutrient intake}–\\ \mathrm{Nutrient in feces} \end{array}\right)/\mathrm{Nutrient intake}\right]\times 100 \end{array}$$

The chemical analyses of the fecal and diets samples included crude protein (CP; method 984.13; AOAC, 2006), ether extract (EE; method 920.39; AOAC, 2006), Ca (method 968.08; AOAC, 2006), and P (method 946.06; AOAC, 2006) as described by AOAC International. The NDF and ADF content was determined using the method of Van Soest et al. (18).

### 2.6 The determination of immune and antioxidant indices

At the end of feeding experiment, one sheep from each replicate with a similar live weight was selected for blood collection. Serum was obtained from non-anticoagulated blood through centrifugation at 3,000 rpm/min for 10 min at 4°C in order to analyze serum biochemical indicators. The concentrations of immunoglobulin M (IgM), immunoglobulin A (IgA), immunoglobulin G (IgG), interleukin 2 (IL-2), interleukin 4 (IL-4), interleukin 6 (IL-6), interleukin 10 (IL-10), interferon-*γ* (IFN-γ), CD4^+^ and CD8^+^ were determined with immunoglobulin-based ELISA kits (Beijing solarbio Bio Tech Company, Shanghai, China). We used the corresponding kit (Shanghai Meilian Bio-Tech Company, Beijing, China) to determine the concentrations of superoxide dismutase (SOD), malondialdehyde (MDA) and glutathione (GSH).

### 2.7 Rumen, hepatic, and jejunum morphology

Following the 60-day experimental period, three Hu sheep of comparable body weight were selected from both groups A and B for slaughter. The goat was weighed with empty stomach in the morning and killed by captive bolt stunning exsanguination. For each sheep, a 1 cm segment of the jejunum was cut with a scalpel, approximately 2 cm × 2 cm of liver tissue, and a 3 cm × 3 cm of the rumen wall were taken. These tissue samples were subsequently washed with saline solution, fixed in 10% formalin buffer for future morphological analysis.

Rumen, hepatic, and jejunum morphologies were detected with hematoxylin–eosin (HE) staining. The specific experimental steps of HE staining refer to the previous study (19, 20), and then observed with a microscope (Olympus Co., Ltd., Tokyo, Japan).

### 2.8 Immunohistochemistry

Immunohistochemical staining was done using the streptavidin-perosidase (SP) method (Thermo Fisher Scientific Company, Shanghai, China). Inflammatory factors (Caspase-3, NF-κB, and TNF-*α*) were detected by immunohistochemistry as described (21). Only the nuclear staining pattern was considered positive. The immunostaining was scored as “0” (negative, no or less than 5% positive cells), “1” (5–25% positive cells), “2” (26–50% positive cells) and “3” (more than 50% positive cells). The staining intensity was scored as follows: 0, colorless; 1, buff; 2, brownish yellow and 3, dark brown. The staining index was obtained by multiplying the staining intensity score by the positive tumor cell score. Based on the heterogeneity of the measure, we defined a staining index of 1–2 as weak, 3–4 as moderate, and 6–9 as strong staining (22, 23).

### 2.9 Rumen fermentation parameters

One sheep from each replicate with a similar live weight was selected for rumen fluid collection. The collection of ruminal fluid was performed 2 h after the morning feeding on day 60 of treatment. Rumen fluid samples were collected through the oral cavity using a rumen tube. To prevent saliva contamination, the initial 50 mL of fluid was discarded, and the next 100 mL was retained. The collected ruminal fluid was then filtered through four layers of cheesecloth. A sample of the ruminal fluid was immediately used to measure pH with a portable pH meter (PHB-4; Shanghai Leici Co., Ltd., Shanghai, China). Additionally, the remaining sample was immediately brought to the laboratory and stored in a − 80°C refrigerator to analyze ruminal bacteria, total and individual VFAs components (acetic acid, propionic acid, and butyric acid), NH_3_-N, protease activity, amylase activity and cellulase activity. Two milliliters of ruminal fluid were immediately stored at −80°C to assess the bacterial population. Ten milliliters of ruminal fluid were added to 2 mL of 25% metaphosphoric acid for assay of VFAs.

Thawed rumen fluid samples were centrifuged at 6000 × g for 10 min, the supernatant collected was analyzed for NH_3_-N based on the protocol described by Ma et al. (24). The concentration of VFAs was determined using crotonic acid as an internal standard by a gas chromatography method (Agilent 7890B, Agilent, California, United States), according to Izuddin et al. (25). The assayed cellulase and amylase activities were measured using the 3,5-dinitrosalicylic acid method (26, 27). Protease activity was determined using the Azocoll assay as previously described (28).

### 2.10 DNA extraction, amplification and Miseq sequencing

Rumen samples were thoroughly homogenized, followed by centrifugation of one milliliter of the rumen fluid for 10 min at 13,200 rpm. The supernatant was discarded, and the remaining pellet was resuspended in 400 mL of nuclease-free water. Microbial DNA extraction was performed utilizing a Genomic DNA Extraction Kit (Takara Biomedical Technology Co., Ltd., Beijing, China) in accordance with the manufacturer’s protocols. After assessing the integrity and purity of DNA, amplification and sequencing were performed as described by Wang et al. (29). The V4–V5 region of the bacteria 16S ribosomal RNA genes was amplified using primers of F:5′-GTGCCAGCMGCCGCGGTAA-3′ and R: 5′-CCGTCAATTCCTTTGAGTTT-3′ with the barcode attached. The concentrations of PCR products were measured using a NanoDrop 2000 spectrophotometer (Thermo Fisher Scientific, Waltham, MA, United States), then merged according to DNA concentration. Purified amplicons were pooled in equimolar and paired-end sequenced (2 × 250) on an Illumina MiSeq platform that was finished by Shanghai Paisennuo Biological Technology Co., Ltd. (Shanghai, China).

### 2.11 Data processing

QIIME2 software package was used to process the raw data. All the raw reads generated from the Illumina sequencing were trimmed using Trimmomatic to remove adapters and low-quality sequences with a phred score of 33 (30). Reads quality control and the resolution of amplicon sequence variants (ASVs) were performed using the DADA2 R package (31). Taxonomic assignment of the ASVs was performed based on the SILVA database (Release132 http://www.Arb-silva.de) (32). Differences in bacterial communities between control and treatment groups were assessed using ANOSIM with the vegan package in R.^1^ Redundancy analysis (RDA) was performed to analyze the correlation between environmental factors and the rumen microbial community. RDA analysis was carried out in reference to the method described by Song et al. (33).

### 2.12 Statistical analysis

All data were analyzed by one-way analysis of variance (ANOVA) using SPSS software (version 22.0; SPSS Institute Inc., Chicago, IL, United States), and the significant differences were compared by Duncan’s multiple range test (*p* < 0.05). The results were expressed as mean values ± standard deviations (mean ± SD).

## 3 Results

### 3.1 Effect of compound postbiotics on the growth performance and economic benefit of *Hu* sheep

The effect of CPs on *Hu* sheep growth performance is presented in Table 2. There was no significant difference for the 30 day body weights of the sheep in all groups. The body weight of 60 day in the groups B and C was higher than that of group A (*p* < 0.05).

**Table 2:** Effects of compound probiotics on the growth performance of *Hu* sheep.

Items	Group A	Group B	Group C	Group D
Initial BW	16.96 ± 0.25	17.14 ± 0.76	17.18 ± 0.55	16.53 ± 0.87
Day 30 BW	21.58 ± 0.63	21.88 ± 0.87	22.03 ± 1.24	21.35 ± 1.32
Final BW	27.04 ± 0.59^b^	28.08 ± 0.45^ab^	28.36 ± 0.62^a^	27.53 ± 1.23^ab^
ADFI (g/d)				
1–30 d	965.18 ± 4.99	962.71 ± 3.39	966.41 ± 2.82	968.89 ± 3.71
31–60 d	1180.85 ± 1.42	1179.98 ± 0.27	1180.85 ± 1.19	1181.69 ± 0.86
1–60 d	1073.30 ± 14.65	1071.00 ± 1.79	1073.63 ± 1.39	1075.29 ± 1.93
ADG (g/d)				
1–30 d	153.73 ± 8.04	158.07 ± 8.17	161.67 ± 10.27	160.67 ± 12.34
31–60 d	182.00 ± 6.81^b^	206.73 ± 15.66^a^	211.07 ± 17.67^a^	206.47 ± 19.55^a^
1–60 d	167.25 ± 13.74^b^	182.20 ± 5.19^a^	186.37 ± 15.08^a^	183.17 ± 10.98^a^
F/G				
1–30 d	6.29 ± 0.33	6.10 ± 0.32	5.97 ± 0.66	6.03 ± 0.94
31–60 d	6.48 ± 0.28^a^	5.71 ± 0.43^b^	5.60 ± 0.49^b^	5.73 ± 0.57^b^
1–60 d	6.42 ± 0.60^a^	5.88 ± 0.18^b^	5.76 ± 0.50^b^	5.88 ± 0.43^b^
Economic benefit (1–60 d)				
Feed intake (kg)	64.40	64.26	64.42	64.25
Feed unit price (yuan/kg)	1.720	1.728	1.736	1.744
Feed cost (yuan/kg)	110.77	111.04	111.83	112.05
Weight gain /kg	10.08	10.94	11.18	11.00
Weight gain income (yuan)	302.40	328.20	335.40	330.00
Economic benefit (yuan/sheep)	191.63	217.16	223.57	217.95

^a, b,^Within a row, means without a common superscript letter differs (*p* < 0.05) or tends to differ (*p* < 0.10) between treatments.

Group A: Control group; Group B: 400 g/t CPs group; Group C: 800 g/t CPs group; Group D: 1200 g/t CPs group.

BW, body weight; ADFI, average daily feed intake; ADG, average daily gain; F/G, the feed to gain ratio.

The price of mutton sheep was calculated according to the average price of local mutton sheep of 30 yuan/kg at beginning of 2023, the compound probiotics cost was 20 yuan/kg, and the economic benefit = weight gain income – feed cost.

The ADFI of *Hu* sheep exhibited no significant differences among the various groups during the pre-period, post-period, and the entire trial period (*p* > 0.05). During days 30 to 60, as well as the entire trial period, the group supplemented with CPs exhibited a higher ADG and a lower F/G compared to group A (control group) (*p* < 0.05). The addition of CPs increased the economic benefit of *Hu* sheep.

### 3.2 Effect of compound postbiotics on the nutrient digestibility of *Hu* sheep

The nutrient digestibility of *Hu* sheep is shown in Table 3. In the early stage, the digestibility of EE in group B was significantly higher compared with the other groups (*p* < 0.05), while no significant differences were observed among the remaining groups. In the post-period, EE digestibility in group D was higher than in groups A, B, and C (*p* < 0.05); The dry matter digestibility of groups B and C was significantly higher than that of group A. During both the pre-trial and post-trial phases, groups B, C, and D exhibited significantly higher Ca digestibility compared with group A (*p* < 0.05).

**Table 3:** Effects of compound probiotics on nutrient digestibility of *Hu* sheep(%).

Items	Group A	Group B	Group C	Group D
Day 30				
DM	73.86 ± 1.15	76.08 ± 1.60	75.81 ± 2.92	75.54 ± 0.72
CP	56.65 ± 3.79	59.53 ± 4.21	57.76 ± 3.34	60.15 ± 2.30
EE	72.16 ± 2.43^b^	75.49 ± 2.55^a^	71.55 ± 2.25^b^	71.64 ± 1.64^b^
NDF	54.18 ± 4.00	57.51 ± 5.08	54.03 ± 3.63	58.44 ± 2.40
ADF	47.48 ± 4.59	50.46 ± 5.92	46.42 ± 4.23	47.50 ± 3.03
Ca	20.30 ± 1.55^b^	32.40 ± 3.66^a^	29.37 ± 3.82^a^	28.39 ± 2.76^a^
P	26.61 ± 1.43	30.61 ± 3.76	30.16 ± 3.78	28.08 ± 0.65
Day 60				
DM	74.38 ± 1.35^b^	78.33 ± 2.01^a^	78.81 ± 1.62^a^	76.79 ± 1.91^ab^
CP	68.31 ± 1.53	68.26 ± 1.65	71.34 ± 1.30	68.28 ± 1.32
EE	77.95 ± 2.38^b^	77.42 ± 2.63^b^	80.10 ± 2.22^b^	83.25 ± 1.85^a^
NDF	60.55 ± 4.26	62.25 ± 4.39	63.94 ± 4.02	62.31 ± 4.16
ADF	50.32 ± 5.37	53.59 ± 5.40	55.41 ± 4.97	56.98 ± 4.74
Ca	23.52 ± 1.49^b^	35.65 ± 3.48^a^	32.43 ± 5.34^a^	35.29 ± 0.59^a^
P	31.79 ± 1.33	38.32 ± 3.34	37.01 ± 5.88	36.83 ± 3.65

^a, b,^Within a row, means without a common superscript letter differs (*p* < 0.05) or tends to differ (*p* < 0.10) between treatments.

Group A: Control group; Group B: 400 g/t CPs group; Group C: 800 g/t CPs group; Group D: 1200 g/t CPs group.

DM, dry matter; CP, crude protein; EE, ether extract; NDF, neutral detergent fiber; ADF, acid detergent fiber.

### 3.3 Effect of compound probiotics on the antioxidant and immune parameters of *Hu* sheep serum

Table 4 showed that the contents of GSH and IgG in group C were significantly higher than that in the other groups (*p* < 0.05). Additionally, the contents of IL-2 and IL-6 in group C were significantly higher than those in group A (*p* < 0.05). The IL-4 level in group D was significantly higher than that in group A (*p* < 0.05); however, no significant difference were observed among groups B, C and D. The content of IFN-*γ* was significantly higher in group C compared to group A (*p* < 0.05); nonetheless, the difference with groups B and D was nonsignificant. The content of CD4^+^ lymphocytes and the CD4^+^/CD8^+^ ratio were highest in group D, but not significantly different from group C.

**Table 4:** Effects of compound probiotics on antioxidant and immunity indexes of *Hu* sheep serum.

Items	Group A	Group B	Group C	Group D
MDA (nmol/mL)	2.64 ± 0.14	2.35 ± 1.01	2.47 ± 0.32	3.05 ± 1.79
SOD (U/mL)	65.85 ± 1.58	66.85 ± 6.70	67.99 ± 5.60	63.39 ± 6.48
GSH (μg/mL)	283.64 ± 46.71^b^	313.37 ± 21.39^b^	442.49 ± 36.94^a^	291.38 ± 11.73^b^
IgG (mg/mL)	109.74 ± 9.91^b^	118.67 ± 7.59^ab^	128.88 ± 7.38^a^	105.74 ± 7.44^b^
IgM (μg/mL)	572.27 ± 48.10	507.12 ± 18.37	563.48 ± 36.82	584.39 ± 56.47
IgA (μg/mL)	454.29 ± 32.77	505.47 ± 44.74	501.21 ± 47.00	501.59 ± 51.00
IL-2 (pg/mL)	1259.93 ± 94.92^b^	1389.64 ± 38.05^ab^	1438.19 ± 73.80^a^	1310.65 ± 105.47^ab^
IL-4 (pg/mL)	70.09 ± 4.86^b^	76.15 ± 4.52^ab^	75.83 ± 1.57^ab^	82.38 ± 9.13^a^
IL-6 (pg/mL)	170.43 ± 18.13^b^	162.96 ± 40.49^b^	222.76 ± 23.84^a^	191.2 ± 33.64^ab^
IL-10 (pg/mL)	213.77 ± 16.60	217.38 ± 4.03	218.95 ± 20.12	213.72 ± 13.49
IFN-γ (pg/mL)	87.56 ± 5.79^b^	95.49 ± 6.53^ab^	110.95 ± 15.78^a^	103.19 ± 6.32^ab^
CD4^+^ (μg/L)	12.19 ± 2.93^b^	17.83 ± 2.17^a^	16.40 ± 1.70^ab^	18.51 ± 2.09^a^
CD8^+^ (μg/L)	7.94 ± 1.33	7.38 ± 0.10	6.81 ± 0.60	7.49 ± 0.71
CD4^+^/CD8^+^	1.52 ± 0.13^b^	2.41 ± 0.27^a^	2.41 ± 0.20^a^	2.50 ± 0.52^a^

^a, b,^Within a row, means without a common superscript letter differs (*p* < 0.05) or tends to differ (*p* < 0.10) between treatments.

Group A: Control group; Group B: 400 g/t CPs group; Group C: 800 g/t CPs group; Group D: 1200 g/t CPs group.

MDA, malondialdehyde; SOD, superoxide dismutase; GSH, reduced glutathione; IgG, immunoglobulin G; IgM, immunoglobulin M; IgA, immunoglobulin A; IL-2, interleukin-2; IL-4, interleukin-4; IL-6, interleukin-6; IL-10, interleukin-10; IFN-γ, interferon-γ.

### 3.4 Effects of compound probiotics on rumen fermentation parameters of *Hu* sheep

Table 5 presents the rumen fermentation parameters, and it showed that the NH_3_-N content was significantly increased in groups B and C compared to group A, with group B exhibiting the highest content (*p* < 0.05). The rumen isobutyrate content in the group B was the highest. The butyric acid content tended to decrease as the amount of CPs added increased, however, the butyric acid content were highest in group A, with no significant differences observed between groups A and B. The amylase activity observed in group C was significantly elevated compared with group A and group D (*p* < 0.05), however, it did not differ significantly from that in group B (*p* < 0.05). Furthermore, group B showed significantly higher cellulase activity compared with all other groups.

**Table 5:** Effects of CPs on rumen fermentation parameters of *Hu* sheep.

Items	Group A	Group B	Group C	Group D
pH	6.68 ± 0.33	6.41 ± 0.31	6.52 ± 0.25	6.47 ± 0.26
Ammoniacal nitrogen (NH_3_-N)(mg/100 mL)	5.50 ± 0.38^c^	8.55 ± 0.81^a^	7.18 ± 0.74^b^	6.08 ± 0.33^c^
Acetic acid(mmol/L)	88.93 ± 12.32	75.10 ± 27.31	56.79 ± 15.99	59.12 ± 6.99
Propionic acid(mmol/L)	19.59 ± 2.70	18.78 ± 7.43	13.38 ± 3.51	13.78 ± 1.89
Isobutyric acid(mmol/L)	1.59 ± 0.23^a^	1.70 ± 0.11^a^	1.25 ± 0.45^ab^	1.13 ± 0.11^b^
Butyric acid(mmol/L)	18.16 ± 2.38^a^	14.87 ± 7.04^ab^	8.97 ± 3.63^b^	8.85 ± 0.34^b^
Protease activity(U/mL)	27.39 ± 7.78	27.82 ± 5.92	34.51 ± 7.21	37.24 ± 9.56
Amylase activity(U/mL)	7.47 ± 0.66^b^	8.01 ± 0.72^ab^	9.78 ± 0.11^a^	3.79 ± 0.93^c^
Cellulase activity(U/mL)	1.63 ± 0.58^b^	2.34 ± 0.02^a^	1.47 ± 0.37^b^	1.44 ± 0.07^b^

^a, b,^Within a row, means without a common superscript letter differs (*p* < 0.05) or tends to differ (*p* < 0.10) between treatments.

Control group; Group B: 400 g/t CPs group; Group C: 800 g/t CPs group; Group D: 1200 g/t CPs group.

NH_3_-N, Ammoniacal nitrogen.

### 3.5 Effects of compound probiotics on rumen microbial diversity of *Hu* sheep

The microbial richness was estimated by observed species and Chao index, the diversity was evaluated by Shannon and Simpson index (Table 6). The Chao index, Shannon index, and the number of observed species in group C were significantly higher than those in group B (*p* < 0.05). Conversely, with the exception of Group C, no significant differences were observed among the other groups (*p* > 0.05).

**Table 6:** Effect of the CPs on alpha diversity of rumen microbiota of *Hu* sheep.

Items	Group A	Group B	Group C	Group D
Observed species	2083.33 ± 260.59^ab^	2013.90 ± 169.47^b^	2351.30 ± 39.62^a^	2089.60 ± 72.72^ab^
Chao1	2477.85 ± 331.03^ab^	2350.75 ± 275.27^b^	2820.81 ± 26.84^a^	2505.96 ± 149.65^ab^
Shannon	9.29 ± 0.30^ab^	9.16 ± 0.28^b^	9.60 ± 0.08^a^	9.46 ± 0.08^ab^
Simpson	0.9948 ± 0.0017	0.9937 ± 0.0032	0.9967 ± 0.0004	0.9965 ± 0.0004

^a, b,^Within a row, means without a common superscript letter differs (*p* < 0.05) or tends to differ (*p* < 0.10) between treatments.

Group A: Control group; Group B: 400 g/t CPs group; Group C: 800 g/t CPs group; Group D: 1200 g/t CPs group.

### 3.6 Gut microbial community influenced by compound probiotics

**Figure 1:**
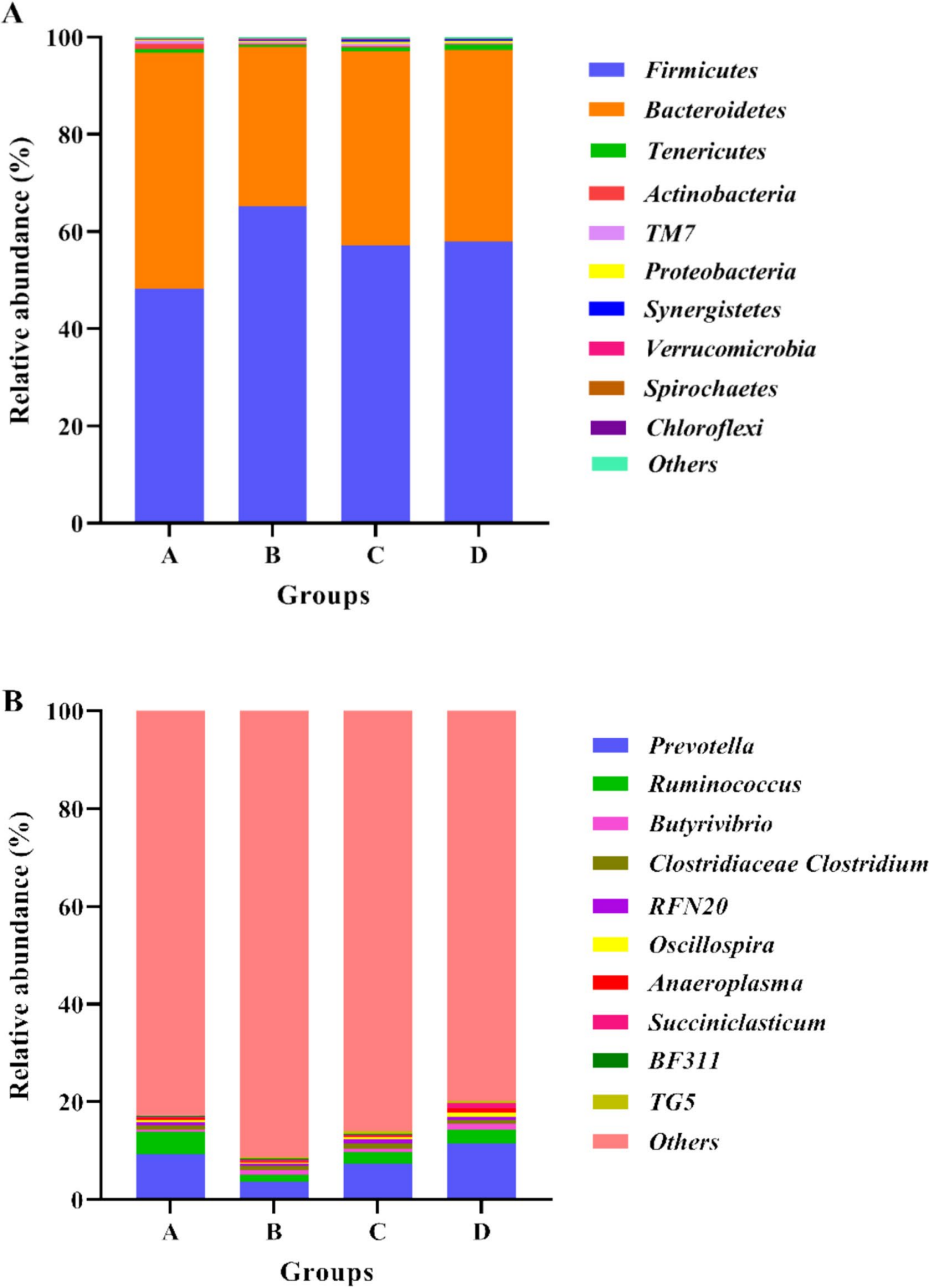
Bacterial compositions and abundances at phylum and genus levels. **(A)** Relative abundance of the top 10 phyla of rumen bacteria; **(B)** Relative abundance of the top 10 genus of rumen bacteria. Group A: Control group; Group B: 400 g/t CPs group; Group C: 800 g/t CPs group; Group D: 1200 g/t CPs group.

Figure 1A showed the abundances of top 10 bacteria at phylum level. The top two major bacterial phyla were *Firmicutes* and *Bacteroidetes*. Compared with group A, the relative abundance of *Firmicutes* increased in the CPs addition groups, whereas the relative abundance of *Bacteroidetes* decreased (Table 7). At the bacterial genus level, the top 10 genera of bacteria are presented in Figure 1B and Table 7. The predominant bacterium in the rumen among the four groups was *Prevotella*, followed by *Ruminococcus* and *Butyrivibrio*. The relative abundance of *Prevotella* and *Anaeroplasma* in group B was lower than that in the other three groups (*p* > 0.05), indicating that the addition of 400 CPs decreased the relative abundance of *Anaeroplasma* and *Prevotella* (Table 7).

**Table 7:** Effects of compound probiotics on the composition and structure of rumen microflora of *Hu* sheep(%).

Items	Group A	Group B	Group C	Group D
Phylum level				
*Firmicutes*	48.17 ± 4.39^b^	65.22 ± 5.32^a^	57.19 ± 0.69^a^	58.01 ± 4.95^a^
*Bacteroidetes*	48.57 ± 3.62^a^	32.70 ± 4.72^b^	39.94 ± 0.23^b^	39.33 ± 4.07^b^
*Tenericutes*	0.82 ± 0.41	0.45 ± 0.15	0.68 ± 0.22	1.14 ± 0.54
*Actinobacteria*	1.12 ± 0.89	0.22 ± 0.32	0.41 ± 0.09	0.18 ± 0.24
*TM7*	0.53 ± 0.34	0.34 ± 0.1946	0.40 ± 0.02	0.20 ± 0.04
*Proteobacteria*	0.21 ± 0.07^b^	0.38 ± 0.05^a^	0.40 ± 0.10^a^	0.32 ± 0.08^ab^
*Synergistetes*	0.05 ± 0.05^b^	0.07 ± 0.05^b^	0.46 ± 0.09^a^	0.35 ± 0.19^a^
*Verrucomicrobia*	0.09 ± 0.11	0.27 ± 0.25	0.10 ± 0.01	0.03 ± 0.03
*Spirochaetes*	0.13 ± 0.07	0.03 ± 0.03	0.09 ± 0.04	0.12 ± 0.07
*Chloroflexi*	0.07 ± 0.09	0.08 ± 0.08	0.06 ± 0.03	0.03 ± 0.02
Genus level				
*Prevotella*	9.34 ± 2.98^a^	3.62 ± 2.67^b^	7.43 ± 0.26^ab^	11.46 ± 1.96^a^
*Ruminococcus*	4.48 ± 2.45	1.44 ± 0.63	2.25 ± 1.18	2.91 ± 0.57
*Butyrivibrio*	0.56 ± 0.366	0.96 ± 0.766	0.81 ± 0.24	1.13 ± 0.35
*Clostridiaceae Clostridium*	0.76 ± 0.226	0.78 ± 0.44	0.93 ± 0.01	0.84 ± 0.26
*RFN20*	0.75 ± 0.06	0.52 ± 0.27	0.98 ± 0.64	0.60 ± 0.26
*Oscillospira*	0.40 ± 0.44	0.35 ± 0.20	0.32 ± 0.13	0.83 ± 0.43
*Anaeroplasma*	0.45 ± 0.20^ab^	0.15 ± 0.08^b^	0.37 ± 0.05^ab^	0.88 ± 0.58^a^
*Succiniclasticum*	0.26 ± 0.32^b^	0.29 ± 0.18^b^	0.22 ± 0.04^b^	1.02 ± 0.32^a^
*BF311*	0.21 ± 0.09	0.42 ± 0.37	0.22 ± 0.07	0.22 ± 0.1
*TG5*	0.05 ± 0.05^b^	0.07 ± 0.05^b^	0.46 ± 0.09^a^	0.35 ± 0.19^a^

^a, b,^Within a row, means without a common superscript letter differs (*p* < 0.05) or tends to differ (*p* < 0.10) between treatments.

Group A: Control group; Group B: 400 g/t CPs group; Group C: 800 g/t CPs group; Group D: 1200 g/t CPs group.

### 3.7 Correlations between rumen bacteria and ruminal fermentation parameters

**Figure 2:**
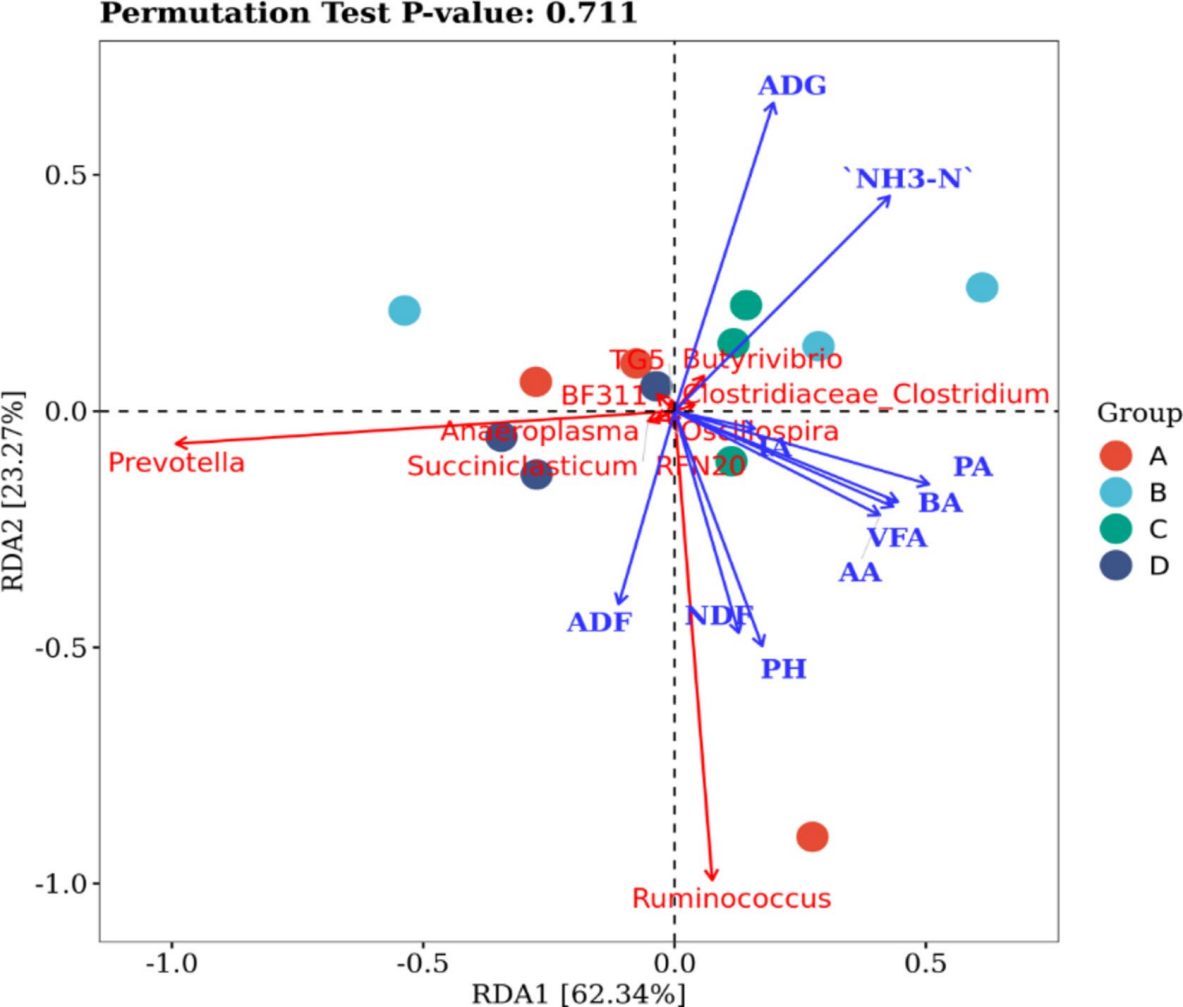
Correlation between bacteria and fermentation parameters in the rumen. VFA is the sum of AA, PA and BA, and NH_3_-N is ammonia nitrogen. AA, acetic acid; PA, propionic acid; BA, butanoic acid.

To investigate the impact of rumen microflora on the progression of rumen fermentation, a correlation analysis was conducted using redundancy analysis (RDA). The results revealed significant correlations between influential rumen microflora at the genus level and ruminal fermentation parameters (Figure 2). *Prevotella* and *Ruminococcus* were strongly correlated with environmental factors. Specifically, *Prevotella* was positively correlated with ADF; *Ruminococcus* was positively correlated with ADF, NDF, pH, acetic acid, propanoic acid, and butyric acid. ADG and NH_3_-N were negatively correlated with both *Prevotella* and *Ruminococcus.*

### 3.8 Effects of compound probiotic on the histological structure of *Hu* sheep

The initial analysis of the results indicated that group B and group C exhibited superior outcomes. However, the difference between the two groups was not significant. Consequently, Group B was selected for further investigation.

**Figure 3:**
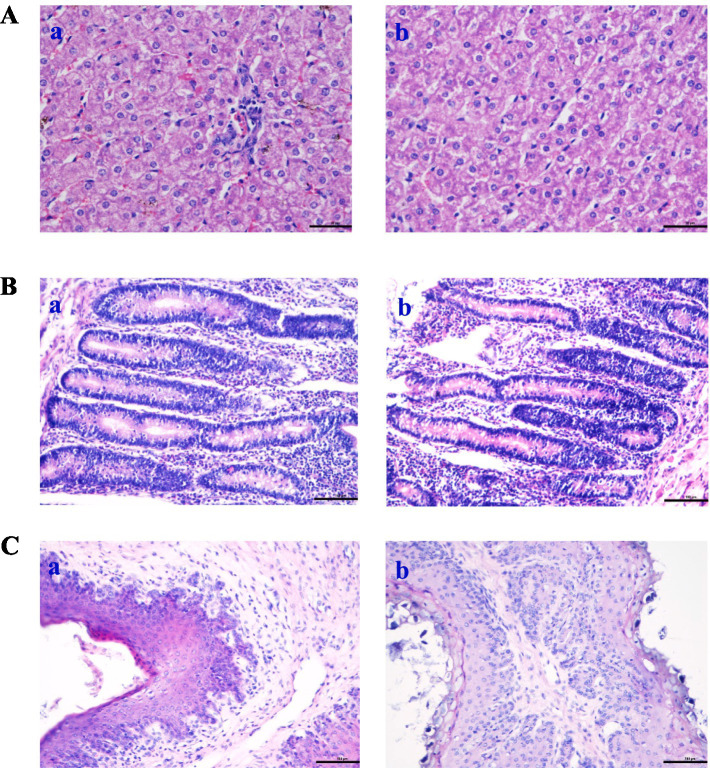
Effects of compound probiotics on the histological structure of *Hu* sheep (×50). **(A)** Liver tissue; **(B)** Jejunum structure; **(C)** Rumen structure. a: the control group (Group A), b: the CPs group (Group B).

Oil Red O staining was performed on liver, jejunum, and rumen sections for groups A and B to verify the digestibility and immunity. The hepatocytes were visualized using HE staining, with signifies the staining of hepatocyte cytoplasm and blue denotes stained nuclei (Figure 3A). The hepatocyte morphology was normal, with hepatic cells being orderly arranged and no abnormalities observed. However, compared to the control group (Figure 3Aa), the cells in the treatment group (Figure 3Ab) exhibited a reduction in inflammatory response and bleeding point. Figure 3B shows the morphology structure of the jejunum. Compared with the control group, group B exhibited a significantly greater number of villi and a higher villus height to crypt depth ratio (Table 8). The analysis of the rumen epithelium revealed that the length and density of the ruminal papillae were significantly increased in the CPs group compared with the control group (Figure 3Cb).

**Table 8:** Effects of compound probiotics on villus height, crypt depth, and villus height/crypt depth ratio of the jejunum of *Hu* sheep.

Items	Group A	Group B	*p*-value
Villus height (μm)	657.96 ± 78.45	737.13 ± 96.01	0.073
Crypt depth (μm)	130.04 ± 10.72	125.44 ± 16.59	0.495
Villus height/Crypt depth	5.08 ± 0.67	5.91 ± 0.65	0.017

Group A: Control group; Group B: 400 g/t CPs group.

### 3.9 Effects of compound probiotics on the inflammatory cytokine expression of *Hu* sheep

**Figure 4:**
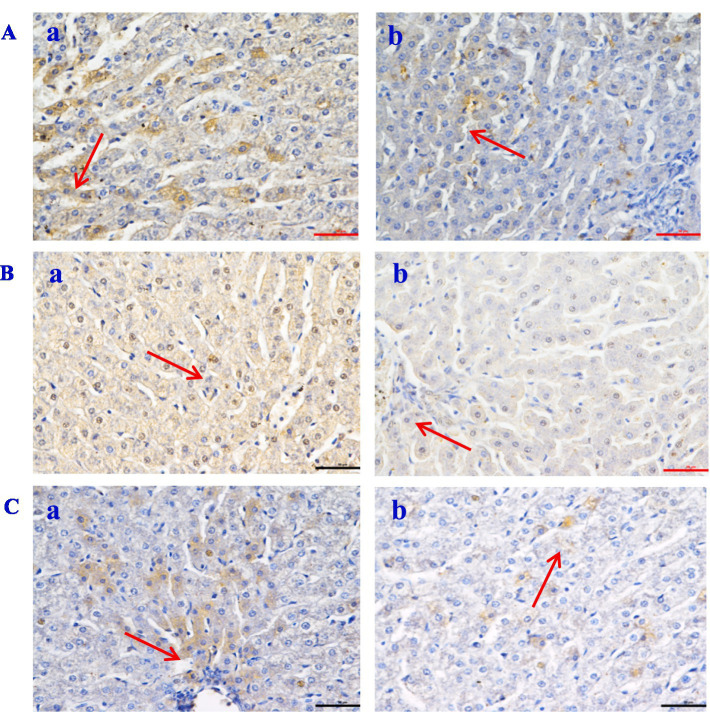
The expression of inflammatory factor in *Hu* sheep liver tissue (×50). **(A)** Caspase-3; **(B)** NF-Κb; **(C)** TNF-*α*. a: the control group (Group A), b: the CPs group (Group B).

**Figure 5:**
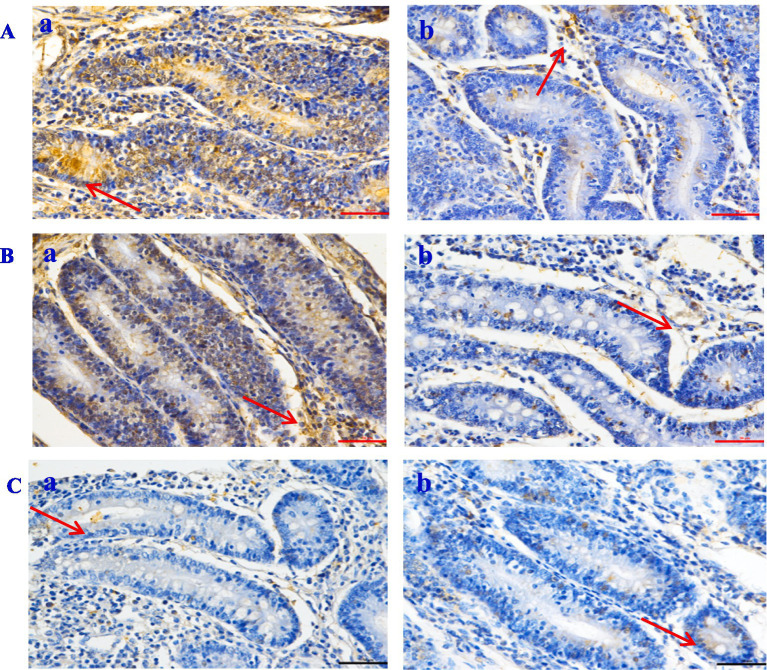
The expression of inflammatory factor in *Hu* sheep jejunal tissue (×50). **(A)** Caspase-3; **(B)** NF-Κb; **(C)** TNF-α. a: the control group (Group A), b: the CPs group (Group B).

**Figure 6:**
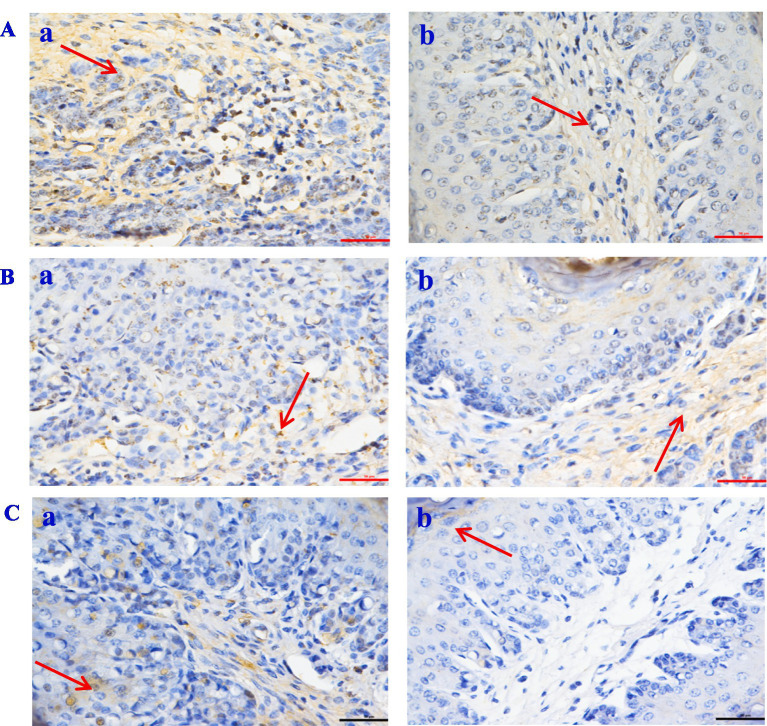
The expression of inflammatory factor in *Hu* sheep rumen tissue (×50). **(A)** Caspase-3; **(B)** NF-Κb; **(C)** TNF-α. a: the control group (Group A), b: the CPs group (Group B).

The histological evaluation (Table 9) of liver, jejunum, and rumen specimens from groups A and B provides evidence supporting the anti-inflammatory activity of CPs. The positive cell detection algorithm measured the number of DAB-positive cells per mm^2^. The control group exhibited a higher number of Caspase-3, NF-κB, and TNF-*α* positive cells, with a darker color, compared with the CPs group. CPs significantly decreased pro-inflammatory factors, including Caspase-3 and NF-κB, in liver and jejunal tissues compared with the control group (Table 9; Figures 4, 5). The immunohistochemistry results indicated that group B had reduced Caspase-3 and TNF-α levels, fewer positive cells, and lighter staining in rumen tissue compared with group A (Table 9; Figure 6).

**Table 9:** Immunohistochemical analysis tissue and immunohistochemistry scores of live, jejunum, and rumen of *Hu* sheep.

Items		Group	Positive cells, %	Score	Positive color	Score	Total score
Liver	Caspase-3	A	20	1	Tan	3	3
		B	10	1	Pale yellow	1	1
	NF-κB	A	70	3	Brown yellow	2	6
		B	80	3	Pale yellow	1	3
	TNF-α	A	30	2	Pale yellow	1	2
		B	10	1	Pale yellow	1	1
Jejunum	Caspase-3	A	50	3	Tan	3	9
		B	10	1	Brown yellow	2	2
	NF-κB	A	40	2	Brown yellow	2	4
		B	10	1	Brown yellow	2	2
	TNF-α	A	5	0	Pale yellow	1	0
		B	5	1	Pale yellow	1	0
Rumen	Caspase-3	A	60	3	Pale yellow	1	3
		B	40	2	Pale yellow	1	2
	NF-κB	A	10	1	Brown yellow	2	4
		B	10	1	Pale yellow	1	1
	TNF-α	A	30	2	Pale yellow	1	3
		B	0	0	Colorless	0	0

## 4 Discussion

Previously, it was shown that the compound probiotics could effectively improve the rumen environment and enhance the intestinal absorptive ability of the nutrients, and improve animal production performance (34–36). The present study showed that the compound probiotics significantly increased the ADG and decreased the F/G during days 31–60 and days 1–60. Guo et al. (8) reported that *Aspergillus oryzae* culture could improve feed dry matter degradation and increase the energy supply of VFAs concentration in the rumen of *Hu* sheep. A previous study revealed that *Aspergillus oryzae* and *Aspergillus niger* co-cultivation extract effectively improved feed digestibility and enhanced bacterial diversity (9). *Candida utilis* could improve feed utilization and intestinal health, consequently enhancing growth performance (37, 38). The present results were consistent with the above studies. However, no significant effects on ADG and F/G were observed during days 1–30, possibly due to the early stage being the colonization period of probiotics in the rumen. The present study indicated that CPs could increase calcium digestibility. Probiotics may influence gut morphology and gene expression to stimulate calcium absorption (39). Studies have indicated that the gut microbiome plays a role in enhancing calcium absorption, increasing calcium retention, and improving various measures of bone health (40), with serum calcium levels improving significantly after probiotic supplementation (41). Probiotics can change the rumen microbial ecosystem in ruminants and promote nutrient digestibility and feed efficiency.

IgM, IgA, and IgG are important immune factors, IgG is the sera’s major immunoglobulin, accounting for approximately 75% of immunoglobulin (42). Studies have shown that many immunoglobulins can improve the non-specific ability of the body’s immunity (43). The present study shows that the serum IgG content increased significantly in group C compared with group A. This result corroborates the findings of previous work on CPs, which can enhance animal immunity and anti-infection ability (44, 45). Strong antioxidant-destroying free radicals were formed when the soybean flour were fermented with *Aspergillus oryzae* (46). In addition, probiotics have been shown to increase GSH levels and reduce intestinal oxidative stress in several experimental models (47, 48). In the study by Lutgendorff et al. (49), it was shown that multispecies probiotics promoted the production of GSH and elevated its levels both in the pancreas and throughout the body. This study is consistent with previous work showing an antioxidant role of CPs. A cytokine is a group of proteins synthesized and secreted by immune and non-immune cells when stimulated (50). In the present study, the levels of cytokines IL-2, IL-4, IL-6, and IFN-*γ* in the serum of experimental sheep from the CPs groups were significantly upregulated compared with the control group. Studies have shown that CD4^+^ T cells are classified into Th1 and Th2 types, which secrete IFN-γ, IL-2 and IL-15 related to cellular immune responses, and IL-4, IL-6, and IL-10 that regulate the production of antibodies (51, 52). Thus, our study implicates that CPs effectively stimulated the Th1 and Th2 type cellular immune responses in sheep. Notably, group C showed the highest levels of both cellular and humoral immunity; however, there was no difference in serum CD4^+^ content among the three levels of CPs groups. The above results suggested that CPs could protect against oxidative stress, improve the body’s immunity, and enhance immune function.

Probiotics have been reported to elevate the efficiency of rumen function by stabilizing rumen pH (25, 53). Study has shown that yeast (*Candida albicans*) can maintain rumen pH stability (54), which was consistent with this study that CPs did not change the rumen pH. The probiotic treatment did not affect the concentrations of major ruminal VFAs, acetic acid and propionic acid in the present study. This agrees with previous reports in which ruminal main VFAs was not affected by a probiotic (55, 56). The levels of butyric acid and isobutyric acid showed a decreasing trend with higher CPs addition (800 CPs and 1,200 CPs). This outcome may be attributed to CPs (Yeast) stimulating the expression of transporters in the rumen epithelium, which could increase the rate of VFAs absorption and thereby decrease in the levels of butyric acid and isobutyric acid (57, 58). The present study showed that the 400 CPs probiotics addition increased the concentration of NH_3_-N, probably due to the reproduction of microorganisms, which increased protein degradation and produced more NH_3_-N (25). *Aspergillus oryzae* 1 and *Aspergillus oryzae* 2 have been proven by our previous research to have the ability to secrete proteases, amylase, and cellulase activity (59, 60). The incorporation of 400 CPs probiotics also improved the amylase and cellulase activity in the rumen, which contributed to improving the digestibility of nutrients.

In this study, the relative abundances of bacteria at the phylum level showed that *Firmicutes* in the CP groups were significantly higher, while *Bacteroidetes* were significantly lower than those in the control group. It was reported that the low ratio between *Bacteroidetes* and *Firmicute*s was beneficial to fat deposition (61), so it can be inferred that CPs addition contributes to sheep body weight gain by decreasing the ratio. At genus levels, the *Prevotella*, *Ruminococcus*, and *Butyrivibrio* were the three dominant genera. The genus *Prevotella* plays an important role in polysaccharide degradation and fermentation in the rumen. Carbohydrates are fermented by *Prevotella* to produce acetic acid (62). This is in keeping with the decrease of acetic acid content in the 400 CPs groups. Furthermore, *Prevotella* and *Ruminococcus* showed a positive correlation with environmental factors. The correlative analysis showed that *Prevotella* was negatively correlated with ADG, while group B with 400 CPs added decreased its relative abundance, indicating CPs increased sheep growth by decreasing *Prevotella* abundance. The specific mechanism needs further study. *Ruminococcus* is a cellulolytic bacterium, producing various cellulolytic enzymes, including cellulase (63, 64). The correlation analysis suggested that there was positive correlation between *Ruminococcus* and NDF. In this study, Group B exhibited the lowest relative abundance of *Ruminococcus*, although this difference was not statistically significant compared with the other groups. Nevertheless, Group B demonstrated the highest cellulase activity, suggesting that the CPs possess the capacity to produce cellulase for the degradation of cellulose.

Intestinal morphology is directly linked to intestinal development and health status (65). The increase in villus height and the ratio of villus height to crypt depth resulted in improved intestinal digestion and absorption efficiency, due to an increase in the absorption area (66). In this experiment, the villus height and the ratio of villus height to crypt depth in the jejunum of sheep fed on CPs were improved compared with the control group. In a study by Yang et al. (37), it was found that *Candida utilis* supplementation increased the villus height of the ileal mucosa and the ratio of villus height to crypt depth while decreasing the crypt depth. These findings suggest that *Candida utilis* has the potential to promote the health of intestinal tissues. This is one of the reasonable explanations for the CPs that can improve the performance and digestibility of the above study. Alkaline protease secreted by *Aspergillus oryzae* has anti-inflammatory activity, which may be useful for treating TNF-*α*-dependent inflammatory bowel diseases (67). TNF-α increases the phagocytic activity of neutrophils and stimulates the inflammatory response (68, 69). In the present study, the number of TNF-α and Caspase-3 positive cells in the liver and rumen in the CPs group was significantly lower than that in the control group. Previous studies have suggested that TNF-α can cause hepatocyte apoptosis via activating the caspase-3 signaling pathway (70, 71); the results of this study are consistent with those previous findings. In this study, the number of NF-κB and Caspase-3 positive cells in the jejunum in the CPs groups was significantly decreased compared with the control group. Furthermore, research has indicated that the NF-κB signaling pathway can activate caspase-3 (72), and there is a positive correlation between the expression of caspase-3 and NF-κB (73). This mechanism reasonably explains the decrease in NF-κB and Caspase-3 observed in the present study. Taken together, these findings suggest that CPs decrease the amount of inflammatory cytokines and the degree of inflammation, thereby reducing cell damage.

## 5 Conclusion

Based on the analysis presented above, it is concluded that adding 800 g/t of composite probiotics to the diet of *Hu* sheep yielded the most favorable outcomes. These included increased daily weight gain, improved feed-to-meat ratio, enhanced immune and anti-inflammatory functions, improved live, rumen and jejunum tissue structure, increased villus height and crypt depth in the jejunum, improved rumen fermentation function, and increased the relative abundance of *Firmicutes* and *Proteobacteria* at the phylum level. In conclusion, the findings of this study indicated the potential utility of compound probiotics in ruminant production.

## Data Availability

The original contributions presented in the study are included in the article/supplementary material, further inquiries can be directed to the corresponding author.

## Footnotes

^1^ https://github.Com/vegandevs/vegan

## References

[1] Melara EG Avellaneda MC Valdivié M García-Hernández Y Aroche R Martínez Y. Probiotics: symbiotic relationship with the animal host. Animals. (2022) 12:719. doi: 10.3390/ani12060719, PMID: 35327116 PMC8944810

[2] Yang M Yin Y Wang F Bao X Long L Tan B. Effects of dietary rosemary extract supplementation on growth performance, nutrient digestibility, antioxidant capacity, intestinal morphology, and microbiota of weaning pigs. J Anim Sci. (2021) 99:skab237. doi: 10.1093/jas/skab237, PMID: 34370023 PMC8420665

[3] Tian S Wang J Yu H Wang J Zhu W. Changes in ileal microbial composition and microbial metabolism by an early-life galacto-oligosaccharides intervention in a neonatal porcine model. Nutrients. (2019) 11:1753. doi: 10.3390/nu11081753, PMID: 31366090 PMC6723927

[4] Kober A Riaz Rajoka MS Mehwish HM Villena J Kitazawa H. Immunomodulation potential of probiotics: a novel strategy for improving livestock health, immunity, and productivity. Microorganisms. (2022) 10:388. doi: 10.3390/microorganisms10020388, PMID: 35208843 PMC8878146

[5] Wei H Geng W Yang XY Kuipers J van der Mei HC Busscher HJ. Activation of a passive, mesoporous silica nanoparticle layer through attachment of bacterially-derived carbon-quantum-dots for protection and functional enhancement of probiotics. Mater Today Bio. (2022) 15:100293. doi: 10.1016/j.mtbio.2022.100293, PMID: 35634173 PMC9130534

[6] Li Y Wang Y Liu Y Li X Feng L Li K. Optimization of an economical medium composition for the coculture of *Clostridium butyricum* and *Bacillus coagulans*. AMB Express. (2022) 12:19. doi: 10.1186/s13568-022-01354-5, PMID: 35166947 PMC8847521

[7] Shruthi B Deepa N Somashekaraiah R Adithi G Divyashree S Sreenivasa MY. Exploring biotechnological and functional characteristics of probiotic yeasts: a review. Biotechnol Rep (Amst). (2022) 34:e00716. doi: 10.1016/j.btre.2022.e00716, PMID: 35257004 PMC8897636

[8] Guo L Zhang D Du R Li F Li F Ran T. Supplementation of aspergillus oryzae culture improved the feed dry matter digestibility and the energy supply of total volatile fatty acid concentrations in the rumen of *Hu* sheep. Front Nutr. (2022) 9:847156. doi: 10.3389/fnut.2022.847156, PMID: 35548561 PMC9084320

[9] Kong F Lu N Liu Y Zhang S Jiang H Wang H. *Aspergillus oryzae* and *aspergillus Niger* co-cultivation extract affects in vitro degradation, fermentation characteristics, and bacterial composition in a diet-specific manner. Animals. (2021) 11:1248. doi: 10.3390/ani11051248, PMID: 33926015 PMC8145302

[10] Hymes-Fecht UC Casper DP. Adaptation and withdrawal of feeding dried *aspergillus oryzae* fermentation product to dairy cattle and goats on in vitro NDF digestibility of selected forage sources. *Transl Anim Sc*i. (2021) 5:txab051. doi: 10.1093/tas/txab051, PMID: 34222819 PMC8244987

[11] Zhang J Jin W Jiang Y Xie F Mao S. Response of milk performance, rumen and hindgut microbiome to dietary supplementation with *aspergillus oryzae* fermentation extracts in dairy cows. Curr Microbiol. (2022) 79:113. doi: 10.1007/s00284-022-02790-z, PMID: 35184209

[12] Miadoková E Svidová S Vlcková V Dúhová V Nad'ová S Rauko P. Diverse biomodulatory effects of glucomannan from *Candida utilis*. Toxicol In Vitro. (2006) 20:649–57. doi: 10.1016/j.tiv.2005.12.001, PMID: 16413741

[13] Vlcková V Dúhová V Svidová S Farkassová A Kamasová S Vlcek D. Antigenotoxic potential of glucomannan on four model test systems. Cell Biol Toxicol. (2004) 20:325–32. doi: 10.1007/s10565-004-0089-7, PMID: 15868477

[14] Yang B Wang D Wei G Liu Z Ge X. Selenium-enriched *Candida utilis*: efficient preparation with l-methionine and antioxidant capacity in rats. J Trace Elem Med Biol. (2013) 27:7–11. doi: 10.1016/j.jtemb.2012.06.001, PMID: 22940082

[15] Chaucheyras-Durand F Ameilbonne A Auffret P Bernard M Mialon MM Dunière L. Supplementation of live yeast based feed additive in early life promotes rumen microbial colonization and fibrolytic potential in lambs. Sci Rep. (2019) 9:19216. doi: 10.1038/s41598-019-55825-0, PMID: 31844130 PMC6914811

[16] Cruz A Håkenåsen IM Skugor A Mydland LT Åkesson CP Hellestveit SS. *Candida utilis* yeast as a protein source for weaned piglets: effects on growth performance and digestive function. Livest Sci. (2019) 226:31–9. doi: 10.1016/j.livsci.2019.06.003

[17] Huang W Chang J Wang P Liu C Yin Q Zhu Q. Effect of the combined compound probiotics with mycotoxin-degradation enzyme on detoxifying aflatoxin B(1) and zearalenone. J Toxicol Sci. (2018) 43:377–85. doi: 10.2131/jts.43.377, PMID: 29877214

[18] Van Soest PJ Robertson JB Lewis BA. Methods for dietary fiber, neutral detergent fiber, and nonstarch polysaccharides in relation to animal nutrition. J Dairy Sci. (1991) 74:3583–97. doi: 10.3168/jds.S0022-0302(91)78551-2, PMID: 1660498

[19] Fei L Sun G Sun J Wu D. The effect of N6-methyladenosine (m6A) factors on the development of acute respiratory distress syndrome in the mouse model. Bioengineered. (2022) 13:7622–34. doi: 10.1080/21655979.2022.2049473, PMID: 35263199 PMC8973778

[20] Song K Zeng X Xie X Zhu R Liang J Chen G. Dl-3-n-butylphthalide attenuates brain injury caused by cortical infarction accompanied by cranial venous drainage disturbance. Stroke Vasc Neurol. (2022) 7:222–36. doi: 10.1136/svn-2021-001308, PMID: 35101948 PMC9240610

[21] Yue D Zhao J Chen H Guo M Chen C Zhou Y. MicroRNA-7, synergizes with RORα, negatively controls the pathology of brain tissue inflammation. J Neuroinflammation. (2020) 17:28. doi: 10.1186/s12974-020-1710-2, PMID: 31959187 PMC6970296

[22] Piao J Shang Y Liu S Piao Y Cui X Li Y. High expression of DEK predicts poor prognosis of gastric adenocarcinoma. Diagn Pathol. (2014) 9:67. doi: 10.1186/1746-1596-9-67, PMID: 24650035 PMC3994479

[23] He W Hou M Zhang H Zeng C He S Chen X. Clinical significance of circulating tumor cells in predicting disease progression and chemotherapy resistance in patients with gestational choriocarcinoma. Int J Cancer. (2019) 144:1421–31. doi: 10.1002/ijc.31742, PMID: 30070688 PMC6587450

[24] Ma T Tu Y Zhang NF Deng KD Diao QY. Effect of the ratio of non-fibrous carbohydrates to neutral detergent fiber and protein structure on intake, digestibility, rumen fermentation, and nitrogen metabolism in lambs. Asian Australas J Anim Sci. (2015) 28:1419–26. doi: 10.5713/ajas.15.0025, PMID: 26323398 PMC4554848

[25] Izuddin WI Loh TC Samsudin AA Foo HL Humam AM Shazali N. Effects of postbiotic supplementation on growth performance, ruminal fermentation and microbial profile, blood metabolite and GHR, IGF-1 and MCT-1 gene expression in post-weaning lambs. BMC Vet Res. (2019) 15:315. doi: 10.1186/s12917-019-2064-9, PMID: 31477098 PMC6719353

[26] Prauchner CA Kozloski GV Farenzena R. Evaluation of sonication treatment and buffer composition on rumen bacteria protein extraction and carboxymethylcellulase activity. J Sci Food Agric. (2013) 93:1733–6. doi: 10.1002/jsfa.5959, PMID: 23180510

[27] Miller GL Blum R Glennon WE Burton AL. Measurement of carboxymethylcellulase activity. Anal Biochem. (1960) 1:127–32. doi: 10.1016/0003-2697(60)90004-X

[28] Elolimy AA Arroyo JM Batistel F Iakiviak MA Loor JJ. Association of residual feed intake with abundance of ruminal bacteria and biopolymer hydrolyzing enzyme activities during the peripartal period and early lactation in Holstein dairy cows. J Anim Sci Biotechnol. (2018) 9:43. doi: 10.1186/s40104-018-0258-9, PMID: 29796256 PMC5956847

[29] Wang L Zhang G Xu H Xin H Zhang Y. Metagenomic analyses of microbial and carbohydrate-active enzymes in the rumen of Holstein cows fed different forage-to-concentrate ratios. Front Microbiol. (2019) 10:649. doi: 10.3389/fmicb.2019.00649, PMID: 30984155 PMC6449447

[30] Bolger AM Lohse M Usadel B. Trimmomatic: a flexible trimmer for illumina sequence data. Bioinformatics. (2014) 30:2114–20. doi: 10.1093/bioinformatics/btu170 24695404 PMC4103590

[31] Callahan BJ McMurdie PJ Rosen MJ Han AW Johnson AJ Holmes SP. DADA2: high-resolution sample inference from illumina amplicon data. Nat Methods. (2016) 13:581–3. doi: 10.1038/nmeth.3869, PMID: 27214047 PMC4927377

[32] Quast C Pruesse E Yilmaz P Gerken J Schweer T Yarza P. The SILVA ribosomal RNA gene database project: improved data processing and web-based tools. Nucleic Acids Res. (2013) 41:D590–6. doi: 10.1093/nar/gks1219, PMID: 23193283 PMC3531112

[33] Song H Singh D Tomlinson KW Yang X Ogwu MC Slik JWF. Tropical forest conversion to rubber plantation in Southwest China results in lower fungal beta diversity and reduced network complexity. FEMS Microbiol Ecol. (2019) 95:fiz092. doi: 10.1093/femsec/fiz092, PMID: 31210262

[34] Lynch HA Martin SA. Effects of *Saccharomyces cerevisiae* culture and *Saccharomyces cerevisiae* live cells on in vitro mixed ruminal microorganism fermentation. J Dairy Sci. (2002) 85:2603–8. doi: 10.3168/jds.S0022-0302(02)74345-2, PMID: 12416814

[35] Uyeno Y Akiyama K Hasunuma T Yamamoto H Yokokawa H Yamaguchi T. Effects of supplementing an active dry yeast product on rumen microbial community composition and on subsequent rumen fermentation of lactating cows in the mid-to-late lactation period. Anim Sci J. (2017) 88:119–24. doi: 10.1111/asj.12612, PMID: 27072297

[36] Sittisak K Pala C Uthai K Rungson S Metha W. Manipulation of rumen ecology by malate and yeast in native cattle. Pak J Nutr. (2009) 8:1048–51. doi: 10.3923/pjn.2009.1048.1051

[37] Yang Z Wang Y He T Ziema Bumbie G Wu L Sun Z. Effects of dietary *yucca schidigera* extract and oral candida utilis on growth performance and intestinal health of weaned piglets. Front Nutr. (2021) 8:685540. doi: 10.3389/fnut.2021.685540, PMID: 34124128 PMC8187599

[38] Shen YB Piao XS Kim SW Wang L Liu P Yoon I. Effects of yeast culture supplementation on growth performance, intestinal health, and immune response of nursery pigs. J Anim Sci. (2009) 87:2614–24. doi: 10.2527/jas.2008-1512, PMID: 19395514

[39] Tiihonen K Ouwehand AC Rautonen N. Human intestinal microbiota and healthy ageing. Ageing Res Rev. (2010) 9:107–16. doi: 10.1016/j.arr.2009.10.004 19874918

[40] Wallace TC Marzorati M Spence L Weaver CM Williamson PS. New frontiers in fibers: innovative and emerging research on the gut microbiome and bone health. J Am Coll Nutr. (2017) 36:218–22. doi: 10.1080/07315724.2016.1257961, PMID: 28318400

[41] Franck A. Prebiotics stimulate calcium absorption: a review. Milchwissenschaft. (2005) 45:11–6. doi: 10.1016/j.nut.2017.06.011

[42] Cheng X He F Si M Sun P Chen Q. Effects of antibiotic use on saliva antibody content and oral microbiota in Sprague dawley rats. Front Cell Infect Microbiol. (2022) 12:721691. doi: 10.3389/fcimb.2022.721691, PMID: 35174102 PMC8843035

[43] Wang Z Zhang F Xiang L Yang Y Wang W Li B. Successful use of extracorporeal life support and continuous renal replacement therapy in the treatment of cardiogenic shock induced by tumor lysis syndrome in a pediatric patient with lymphoma: a case report. Front Med. (2021) 8:762788. doi: 10.3389/fmed.2021.762788, PMID: 35059412 PMC8764359

[44] Xu X Yang C Chang J Wang P Yin Q Liu C. Dietary supplementation with compound probiotics and berberine alters piglet production performance and fecal microbiota. Animals. (2020) 10:511. doi: 10.3390/ani10030511, PMID: 32204369 PMC7142521

[45] Liu X Zhao W Yu D Cheng JG Luo Y Wang Y. Effects of compound probiotics on the weight, immunity performance and fecal microbiota of forest musk deer. Sci Rep. (2019) 9:19146. doi: 10.1038/s41598-019-55731-5, PMID: 31844127 PMC6915770

[46] Handa CL de Lima FS Guelfi MFG Fernandes MDS Georgetti SR Ida EI. Parameters of the fermentation of soybean flour by Monascus purpureus or *aspergillus oryzae* on the production of bioactive compounds and antioxidant activity. Food Chem. (2019) 271:274–83. doi: 10.1016/j.foodchem.2018.07.188 30236677

[47] Tang Y Han L Chen X Xie M Kong W Wu Z. Dietary supplementation of probiotic *Bacillus subtilis* affects antioxidant defenses and immune response in grass carp under *aeromonas hydrophila* challenge. Probiotics Antimicrob Proteins. (2019) 11:545–58. doi: 10.1007/s12602-018-9409-8, PMID: 29654472

[48] Peran L Camuesco D Comalada M Nieto A Concha A Adrio JL. *Lactobacillus fermentum*, a probiotic capable to release glutathione, prevents colonic inflammation in the TNBS model of rat colitis. Int J Color Dis. (2006) 21:737–46. doi: 10.1007/s00384-005-0773-y, PMID: 16052308

[49] Lutgendorff F Trulsson LM van Minnen LP Rijkers GT Timmerman HM Franzén LE. Probiotics enhance pancreatic glutathione biosynthesis and reduce oxidative stress in experimental acute pancreatitis. Am J Physiol Gastrointest Liver Physiol. (2008) 295:G1111–21. doi: 10.1152/ajpgi.00603.2007, PMID: 18832452

[50] Zhang J Zhang Y Wang Q Li C Deng H Si C. Interleukin-35 in immune-related diseases: protection or destruction. Immunology. (2019) 157:13–20. doi: 10.1111/imm.13044, PMID: 30681737 PMC6459776

[51] Toda M Ono SJ. Genomics and proteomics of allergic disease. Immunology. (2002) 106:1–10. doi: 10.1046/j.1365-2567.2002.01407.x, PMID: 11972626 PMC1782693

[52] Kanai K Asano K Watanabe S Kyo Y Suzaki H. Epinastine hydrochloride antagonism against interleukin-4-mediated T cell cytokine imbalance in vitro. Int Arch Allergy Immunol. (2006) 140:43–52. doi: 10.1159/000092001, PMID: 16534218

[53] O'Callaghan TF Ross RP Stanton C Clarke G. The gut microbiome as a virtual endocrine organ with implications for farm and domestic animal endocrinology. Domest Anim Endocrinol. (2016) 56:S44–55. doi: 10.1016/j.domaniend.2016.05.003, PMID: 27345323

[54] Liu Y Xiao Y Ma T Diao Q Tu Y. *Candida tropicalis* as a novel dietary additive to reduce methane emissions and nitrogen excretion in sheep. Environ Sci Pollut Res Int. (2023) 30:82661–71. doi: 10.1007/s11356-023-28245-x, PMID: 37329373

[55] Chiquette J Allison MJ Rasmussen MA. *Prevotella bryantii* 25A used as a probiotic in early-lactation dairy cows: effect on ruminal fermentation characteristics, milk production, and milk composition. J Dairy Sci. (2008) 91:3536–43. doi: 10.3168/jds.2007-0849, PMID: 18765612

[56] Ghorbani GR Morgavi DP Beauchemin KA Leedle JA. Effects of bacterial direct-fed microbials on ruminal fermentation, blood variables, and the microbial populations of feedlot cattle. J Anim Sci. (2002) 80:1977–85. doi: 10.2527/2002.8071977x, PMID: 12162668

[57] Shah AM Cai Y Zou H Zhang X Wang L Xue B. Effects of supplementation of branches and leaves trimmed from tea plant on growth performance, rumen fermentation and meat composition of nanjiang yellow goats. Animals. (2019) 9:590. doi: 10.3390/ani9090590 31438584 PMC6769452

[58] Petri RM Wetzels SU Qumar M Khiaosa-Ard R Zebeli Q. Adaptive responses in short-chain fatty acid absorption, gene expression, and bacterial community of the bovine rumen epithelium recovered from a continuous or transient high-grain feeding. J Dairy Sci. (2019) 102:5361–78. doi: 10.3168/jds.2018-15691, PMID: 31005320

[59] Chang J Cheng W Yin Q Zuo R Song A Zheng Q. Effect of steam explosion and microbial fermentation on cellulose and lignin degradation of corn Stover. Bioresour Technol. (2012) 104:587–92. doi: 10.1016/j.biortech.2011.10.070, PMID: 22104102

[60] Chang J Wang T Wang P Yin Q Liu C Zhu Q. Compound probiotics alleviating aflatoxin B(1) and zearalenone toxic effects on broiler production performance and gut microbiota. Ecotoxicol Environ Saf. (2020) 194:110420. doi: 10.1016/j.ecoenv.2020.110420, PMID: 32151861

[61] Turnbaugh PJ Ley RE Mahowald MA Magrini V Mardis ER Gordon JI. An obesity-associated gut microbiome with increased capacity for energy harvest. Nature. (2006) 444:1027–31. doi: 10.1038/nature05414, PMID: 17183312

[62] Shinkai T Ikeyama N Kumagai M Ohmori H Sakamoto M Ohkuma M. *Prevotella* lacticifex sp. nov., isolated from the rumen of cows. Int J Syst Evol Microbiol. (2022) 72:5278. doi: 10.1099/ijsem.0.005278, PMID: 35254232

[63] Kawahara R Saburi W Odaka R Taguchi H Ito S Mori H. Metabolic mechanism of mannan in a ruminal bacterium, *Ruminococcus albus*, involving two mannoside phosphorylases and cellobiose 2-epimerase: discovery of a new carbohydrate phosphorylase, β-1,4-mannooligosaccharide phosphorylase. J Biol Chem. (2012) 287:42389–99. doi: 10.1074/jbc.M112.390336, PMID: 23093406 PMC3516782

[64] Zhang XM Medrano RF Wang M Beauchemin KA Ma ZY Wang R. Corn oil supplementation enhances hydrogen use for biohydrogenation, inhibits methanogenesis, and alters fermentation pathways and the microbial community in the rumen of goats. J Anim Sci. (2019) 97:4999–5008. doi: 10.1093/jas/skz352, PMID: 31740932 PMC6915217

[65] Huang D Zhu J Zhang L Ge X Ren M Liang H. Dietary supplementation with eucommia ulmoides leaf extract improved the intestinal antioxidant capacity, immune response, and disease resistance against *streptococcus agalactiae* in genetically improved farmed tilapia (GIFT; *Oreochromis niloticus*). Antioxidants. (2022) 11:1800. doi: 10.3390/antiox11091800, PMID: 36139874 PMC9495437

[66] Sun H Tang JW Yao XH Wu YF Wang X Feng J. Effects of dietary inclusion of fermented cottonseed meal on growth, cecal microbial population, small intestinal morphology, and digestive enzyme activity of broilers. Trop Anim Health Prod. (2013) 45:987–93. doi: 10.1007/s11250-012-0322-y, PMID: 23224950

[67] Hong K Yu J Parmely MJ. Alkaline protease purified from aspergillus oryzae prevents TNF-a-induced acute inflammation in the mouse small intestine. FASEB J. (2006) 20:A144. doi: 10.1096/fasebj.20.4.A144

[68] Carmona-Rivera C Khaznadar SS Shwin KW Irizarry-Caro JA O'Neil LJ Liu Y. Deficiency of adenosine deaminase 2 triggers adenosine-mediated NETosis and TNF production in patients with DADA2. Blood. (2019) 134:395–406. doi: 10.1182/blood.2018892752, PMID: 31015188 PMC6659253

[69] Matte A Recchiuti A Federti E Koehl B Mintz T El Nemer W. Resolution of sickle cell disease-associated inflammation and tissue damage with 17R-resolvin D1. Blood. (2019) 133:252–65. doi: 10.1182/blood-2018-07-865378, PMID: 30404812 PMC6337877

[70] Guerra AD Yeung OWH Qi X Kao WJ Man K. The anti-tumor effects of M1 macrophage-loaded poly (ethylene glycol) and gelatin-based hydrogels on hepatocellular carcinoma. Theranostics. (2017) 7:3732–44. doi: 10.7150/thno.20251, PMID: 29109772 PMC5667344

[71] Wang S Li L Shi L. Identification of a key candidate gene-phenotype network mediated by glycyrrhizic acid using pharmacogenomic analysis. Mol Med Rep. (2019) 20:2657–66. doi: 10.3892/mmr.2019.10494, PMID: 31322195 PMC6691250

[72] Milani A Basirnejad M Bolhassani A. Heat-shock proteins in diagnosis and treatment: an overview of different biochemical and immunological functions. Immunotherapy. (2019) 11:215–39. doi: 10.2217/imt-2018-0105, PMID: 30730280

[73] Zhu X Wu Y Li C Yan W Pan J Wang S. Prenatal exposure to gossypol impairs Corticogenesis of mouse. Front Neurosci. (2020) 14:318. doi: 10.3389/fnins.2020.00318, PMID: 32317927 PMC7146080

